# Editorial: Quality of Horticultural Crops: A Recurrent/New Challenge for Plant Scientists in a Changing World

**DOI:** 10.3389/fpls.2018.01151

**Published:** 2018-08-09

**Authors:** Nadia Bertin, Michel Génard, Maarten Hertog

**Affiliations:** ^1^Plantes et Systèmes de Culture Horticoles, INRA, Avignon, France; ^2^Department of Biosystems, KU Leuven, Leuven, Belgium

**Keywords:** quality, horticultural crops, health value, fruit, vegetable, pre- post-harvest links

Improving crop quality is a challenge in the context of a global horticultural food supply, since the development of sustainable crop production systems inevitably affects many quality traits. Fruit and vegetable quality includes size, visual attractiveness (color, shape), overall flavor (taste and texture), health benefits, shelf life, suitability for processing…etc. At each step of the production chain, specific criteria prevail depending on the product's final destination, either the fresh market or the processing industry. These criteria are not necessarily the same throughout the chain, and likely interact during the product's life. Thus, the management and improvement of postharvest quality requires the integration of knowledge from the field until purchase and consumption of the fresh or processed product. This e-collection collates state-of-the-art research outputs on the quality of fruits and vegetables from seed to fork, covering the underlying physiological processes, the genetic and environmental controls during plant and organ development and the postharvest evolution of quality during storage and processing.

## Molecular controls behind quality traits

The molecular and genetic controls behind quality build-up are illustrated on different species. Diouf et al. emphasized the complexity of the interaction between genotype and environment in controlling plant growth and fruit quality traits. Based on the MAGIC Tomato population, these authors showed that 33–86% of the phenotypic variation in yield and quality traits is due to the genetics. Among the 54 QTLs detected, 15 revealed significant interactions between genotype and water or salinity stress and 35 QTLs were treatment specific. In citrus, which is an important fruit crop in Mediterranean regions, comparative transcriptome analysis showed that starch and GA metabolisms in peel are involved in roughing disorder induced by severe fruit thinning (Lu et al.). Miao et al. performed a functional analysis of soluble starch synthase genes during banana development and storage and they identified MaSSIII-1 as a key gene responsible for amylopectin biosynthesis. In citrus, molecular events associated with low temperature tolerance induced by heat-conditioning open new perspectives to reduce chilling injury (Lafuente et al.).

## Main quality traits

Color and pigment accumulation are major criteria of fruit and vegetable quality, which are addressed in several papers. Muleke et al. studied the spatial and temporal regulation of anthocyanin biosynthesis genes in radish taproots and identified five candidate genes that play a major role in phenotypic variations. In red-fleshed kiwi, Li et al. characterized genes involved in the increase of anthocyanin biosynthesis and accumulation during postharvest storage. In pear cultivars differing in color fading, Wang et al. analyzed differentially expressed genes linked to a late decrease in anthocyanin biosynthesis, and which increased during anthocyanin degradation in peel, suggesting the involvement of light signals. Feng et al. evidenced the role of MdMADS1, a MADS-Box gene, in ALA (a plant growth regulator)-induced anthocyanin accumulation in apple skin. Karagiannis et al. observed higher pigmentation of peach skin grown at high altitude and discussed the altitude effect on protein variations potentially involved in ripening. Jia et al. evidenced the role of FERONIA-like receptor kinase in the control of strawberry ripening by modulating ABA signaling.

Aroma is another important and complex trait of fruit quality. Farneti et al. explored the blueberry volatile organic compounds during ripening by two profiling methods and proposed biomarkers of berry physiology that could be used to phenotype genetic resources. Testone et al. showed that the regulation of sesquiterpene lactone biosynthesis, that confers bitterness to stem chicory, occurs at the transcriptional level.

Regarding the accumulation of health related compounds, Wiesner-Reinhold et al. emphasized the role of selenium and sulfur metabolisms in Brassicaceae for human health and reviewed some works on biofortification of this plant family. More generally, Zhang et al. examined the links between ploidy level and agronomical traits and observed some antagonisms among quality traits of kiwi berries, for instance between vitamin C and sugar contents which were, respectively higher and lower in germplasms with high ploidy level, thus questioning the overall advantage of high ploidy.

## The impact of preharvest stresses

Quality variations induced by water and salinity stress are important issues in horticultural crops. At the fruit scale, Miras-Avalos, and Intrigliolo made a meta-analysis of negative and positive effects of these stresses on grape composition and final wine quality, and they proposed a statistical model accounting for the agronomical context. In olive trees, the effects of the watering regime on the molecular and biochemical mechanisms that regulate the accumulation of main phenolic compounds during drupe development, were reported by Cirilli et al., who suggest a differential sensitivity of enzymes involved in phenolic catabolism. At the orchard scale, the study of Käthner at al. based on a thermal imaging approach to evaluate crop water stress index (CWSI) and soil electrical conductivity analysis, revealed that fruit quality could be predicted by interactions between CWSI and cumulative water use efficiency. Grafting is a key-alternative to adapt to environmental constraints. Kyriacou et al. reviewed how grafting, a practice largely developed for Curcubitaceae and Solanaceae production, influences fruit quality and storability.

## Preharvest impact on postharvest quality

During the postharvest period, as maturation progresses, genetic, chemicals and environmental control can help maintain product quality. Improving shelf life while reducing the use of chemicals is obligatory to meet the consumers' demand and reduce losses along the food chain. Regarding products intended for processing, technological quality traits such as color, texture, viscosity or bio-accessibility impact on the final quality. However, the link between quality build-up in the preharvest period and its impact on the technological quality traits have been largely overlooked and detailed knowledge is still missing to really bridge this gap. Alamar et al. reviewed some alternative methods to control sprouting and diseases to ensure potato tuber quality, stressing the need to better understand interactions between pre- and postharvest factors. As such, Arbex de Castro Vilas Boas et al. evidenced significant interactions among genotype, preharvest interventions and processing methods, affecting major traits of both the fruit and the processed products derived from it, suggesting that managing the quality of processed products starts in the field. The moment of harvest is known to have a clear impact on postharvest quality traits. In blueberry, Moggia et al. observed that soft fruits at harvest exhibited higher softening rate and internal browning as compared to firm fruit cultivars, whereas mechanical damage induced variations in storability of the firm fruit cultivar only.

Finally, modeling the interactions among the factors that control quality in the pre and postharvest fruit life has proven its interest to optimize crop management and understand genetic variability. Tran et al. proposed a model-based optimization approach to optimize harvest date and frequency for field tomato, by predicting fruit-to-fruit variation in ripening and the associated crop economic value.

All these works do contribute in their own right to the complex answer on how to meet the future demand for better quality food in a changing environment, where resources will soon become limited.

## Author contributions

All authors listed have made a substantial, direct and intellectual contribution to the work, and approved it for publication.

### Conflict of interest statement

The authors declare that the research was conducted in the absence of any commercial or financial relationships that could be construed as a potential conflict of interest.

